# Evaluation of the Viscoelastic Properties of Lower-Extremity Muscles of Pediatric Hemophilia Patients Using Myotonometric Measurements

**DOI:** 10.3390/children11020229

**Published:** 2024-02-09

**Authors:** Tuğba Gönen, Serkan Usgu, Yavuz Yakut, Sinan Akbayram

**Affiliations:** 1 Department of Physical Therapy and Rehabilitation, Faculty of Health Sciences, Hasan Kalyoncu University, Gaziantep 27000, Turkey; tugba.badat@hku.edu.tr (T.G.); yavuz.yakut@hku.edu.tr (Y.Y.); 2 Department of Pediatric Hematology-Oncology, Faculty of Medicine, Gaziantep University, Gaziantep 27310, Turkey; sinanakbayram@gantep.edu.tr

**Keywords:** hemophilia, tone, stiffness, elasticity, hemarthrosis, hematoma

## Abstract

This study aimed to evaluate the viscoelastic properties of lower-extremity muscles in pediatric hemophilia (FVIII-IX) patients. The study included 20 severe- and moderate-type right-dominant hemophilia patients diagnosed with hemophilia A–B and 20 healthy children. Viscoelastic properties (tone, stiffness, elasticity) of the lower-extremity muscles were measured using a MyotonPRO device. The physical characteristics of the pediatric hemophilia patients (mean age: 11.9 ± 3.95 years) and the control group (mean age: 12.6 ± 3.41 years) were found to be similar. A difference was observed only in the elasticity of the right vastus lateralis (*p* < 0.05) by means of the viscoelastic properties of the lower-extremity muscles. The results were similar in other muscle groups (*p* > 0.05). The dominant-side vastus lateralis muscle elasticity (the ability of the muscle to regain its original shape after contraction or removal of an external force) of hemophilia patients was found to be lower compared to healthy children. The fact that 45% of hemarthroses occur in the knee joint and that recurrent bleeding may affect the flexibility of the vastus lateralis, which is the main muscle within the quadriceps muscle group and responsible for the stabilization of the patella, can be associated with the study results.

## 1. Introduction

Hemophilia A and hemophilia B are congenital bleeding disorders caused by the deficiency or absence of one of two clotting proteins: factor VIII and factor IX. Hemophilia A and B affect only boys because they are X-linked recessive diseases. Hemophilia severity is classified as severe, moderate, or mild according to the level of the factor present in the body: severe (<1 (IU)/dL), moderate (1–5 IU/dL), mild (6 IU/dL to <40 IU/dL) [1]. Hemarthrosis and hematomas occurring in children with severe and moderate hemophilia are the most typical features of the disease. Hemarthroses (80%) due to bleeding within the joint and hematomas (20%) due to intramuscular bleeding cause musculoskeletal system problems such as joint degeneration and muscle atrophy [2]. Physiological changes that occur in the muscle after a history of bleeding affect muscle tone and decrease stiffness. It is important to evaluate changes in the mechanical properties of muscles that may limit physical function and activities of daily living [3,4]. Often, technological devices such as electromyography and elastography are used to evaluate the viscoelastic properties of the muscle [5]. Electromyography (EMG) is one of the best-known and widely used methods to objectively assess muscle function [6]. Other methods such as elastography, shear wave ultrasound elastography, or free oscillation techniques are also valid and reliable tools to measure the mechanical properties of muscles and tendons [7,8]. However, accessing these systems in clinics requires high costs and experience. Considering studies conducted on healthy individuals, it has been shown that there is a strong relationship between shear wave ultrasonography on various muscle groups and measurements of muscle mechanical properties using MyotonPRO [9]. For this reason, the inexpensive and user-friendly MyotonPRO (Müomeetria Ltd., Tallinn, Estonia) tool, which was created to assess the mechanical characteristics of the musculoskeletal system, is a legitimate and trustworthy technique that offers impartial information to assess the tone, stiffness, and elasticity of muscles [10]. MyotonPRO is a small, non-invasive handheld device that can transport data without requiring a computer connection during data collection. Its ease of use and suitability for clinical testing make it a desirable option. By percutaneously delivering a quick-release mechanical hit to the muscle, it induces oscillation in the muscle. By measuring the oscillation occurring in the muscle with an accelerometer, parameters related to mechanical properties can be calculated simultaneously [11]. MyotonPRO has been used to evaluate mechanical properties in healthy individuals [12], athletes [13], and geriatric individuals [14], and various diseases [15,16]. However, in the pediatric group, myotonometric measurements are frequently preferred in diseases such as cerebral palsy, brachial plexus injury, and in children with developmental delays [17,18,19,20]. To date, no study has been found to investigate the changes in the viscoelastic properties of the lower-extremity muscles in pediatric hemophilia patients. This study aimed to evaluate the viscoelastic properties of lower-extremity muscles in pediatric hemophilia (FVIII-IX) patients with myotonometric measurements and compare them with their healthy peers.

## 2. Materials and Methods

### 2.1. Participants

Our cross-sectional planned study was conducted to evaluate the viscoelastic properties (tone, stiffness, elasticity) of the lower-extremity muscles in pediatric patients diagnosed with hemophilia A and hemophilia B with myotonometric measurements, and compare them with their healthy peers. Patients who were followed up with a diagnosis of hemophilia A or hemophilia B at the Pediatric Hematology Polyclinic of Gaziantep University Sahinbey Application and Research Hospital participated in the study conducted at Hasan Kalyoncu University Faculty of Health Sciences Department of Physiotherapy and Rehabilitation. Our study received ethical approval number 2023/52 from Hasan Kalyoncu University Health Sciences Non-Invasive Research Ethics Committee on 22 May 2023. Additionally, our study was registered in the International Registry (ID: NCT05981313).

In total, 20 hemophilia patients diagnosed with severe- or moderate-type hemophilia A (*n* = 13) or hemophilia B (*n* = 7), aged between 6 and 17, and 20 healthy children were included in the study. All children included in the study were right-dominant and male.

The inclusion criteria were as follows:

- Patients who did not have a history of acute bleeding in the lower extremities;
- Receiving regular prophylaxis treatment;
- Consent obtained from parents of children who volunteered to participate in the study.

The exclusion criteria were as follows:

- Patients with a history of lower-extremity surgery;
- Neurological disease;
- Patients with a history of lower-extremity hemarthrosis or hematoma within the last month.

### 2.2. Measurements

Myotonometric measurements were performed by a physiotherapist experienced in the field of hemophilia. First, the children’s demographic information, such as name, surname, age, height, weight, and body mass index (BMI), was taken and recorded in the “Data Collection Form”. In addition, the hemophilia type and severity, factor levels, medication use, presence of target joint in the lower extremity, and arthropathy findings of the patients in the hemophilia group were evaluated.

The muscles measured in the lower extremity were as follows:

1. M. vastus medialis obliquus (VMO);
2. M. rectus femoris (RF);
3. M. vastus lateralis (VL);
4. M. biceps femoris (BF);
5. M. tibialis anterior (TA);
6. M. gastrocinemius (GM, GL).

**Figure 1:**
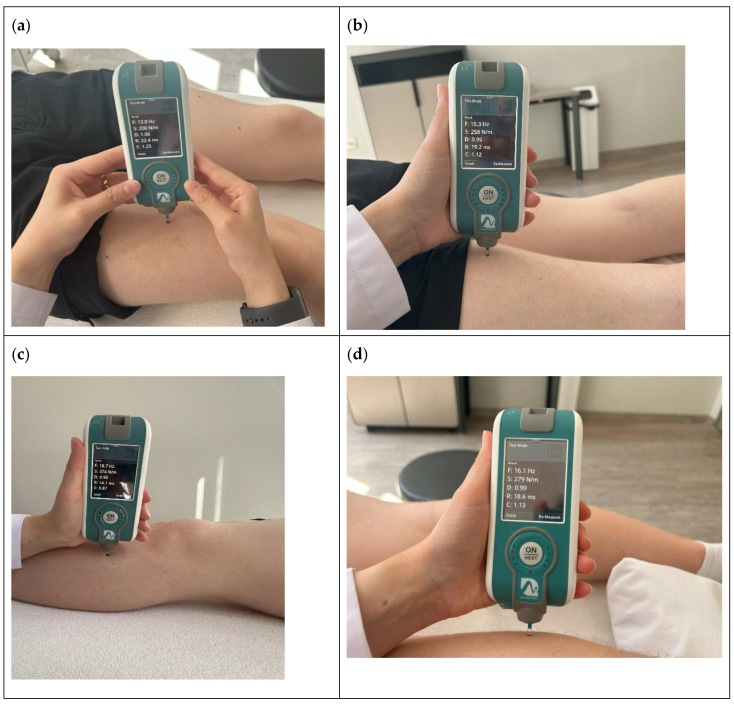
Examples of muscles reference points for myotonometric assessment: (**a**) rectus femoris; (**b**) biceps femoris; (**c**) tibialis anterior; (**d**) gastrocinemius.

M. vastus medialis from the most swollen point of the obliquus muscle, M. rectus femoris from the 2/3 proximal between the spina iliaca anterior superior (CIAS) and the patella, M. vastus lateralis from the 2/3 proximal between the major trochanter and the lateral condyle, and M. tibialis anterior from 12 cm below the lateral condyle of the femur on the back were evaluated in the relaxed position. M. biceps femoris and M. gastrocinemius measurements were taken in a relaxed prone position, while M. biceps femoris was measured from the midpoint of the tuberischiadicum and popliteal fossa and M. gastrocinemius measurements were taken from the most swollen point proximally, with the ankle in neutral position for the medial and lateral head [5,21,22] (Figure 1).

Afterwards, the viscoelastic properties (tone, stiffness, elasticity) of the lower-extremity muscles were measured from determined reference points using the MyotonPRO device. Participants were allowed to relax at the table for 10 min before measurements were taken. The reference points of the relevant muscles were marked with a pencil for ease of subsequent measurements. The point is to obtain the correct orientation by contacting the probe with the skin among the inter-raters. The device only takes measurements when it is held perpendicular to the surface, and when the positioning is incorrect, a red light and the word “Rotate” appear on the screen. When the probe contacts the skin, the green light turns on when the probe is placed vertically on the correct axis for measurement. The mechanism ensures the reliability of the repeatability of measurement among inter-raters. Viscoelastic measurement was repeated three times from the reference points determined by ensuring correct orientation and the averages were recorded. Only measurements with a co-efficient of variation (CV) less than 3% were taken into account, and when the CV was higher than 3%, the measurement was repeated 5 times for the same reference point [23]. Measurements were taken by the same physiotherapist at noon. We aimed to ensure similar environmental conditions on each measurement day, taking into account room temperature and humidity conditions (temperature (22/24 C); humidity (mean; 50%)).

#### MyotonPRO

The measurement method of the device is based on the free oscillation technique. The device enables the simultaneous calculation of muscle mechanical properties. Tone, which is the frequency of oscillation (Hz), is defined as resting tension or resistance to passive stretching at rest. Frequency is the maximum frequency calculated from the signal spectrum by FFT (fast Fourier transform (F = fmax)). The higher the natural oscillation frequency, the higher the tone. The frequency increases with contraction and stretching. Stiffness (N/m) is defined as the ability to resist force that changes its shape. Stiffness is calculated as s = m_probe_ (a_max_/Δl) and the higher the N/m value, the greater the muscle stiffness [24]. Elasticity (logarithmic decrease) (log) refers to the ability of the muscle to regain its shape after being deformed. Elasticity is expressed in arbitrary units and indicates how much mechanical energy is lost in the tissue during one cycle of oscillation. The less reduction value, the less dissipation of mechanical energy, and therefore the higher the elasticity of a tissue will be [25].

### 2.3. Statistical Analysis

The SPSS version 22.0 (IBM Corp., Armonk, NY, USA) package program was used to analyze the data. Descriptive statistics were summarized as arithmetic mean and standard deviation (X ± SD) for numerical variables, and frequency and percentage for categorical variables. Normal distribution of the data was determined by Kolmogorov–Smirnov analysis and frequency histograms. For normally distributed data, an unpaired *T*-test was used to compare the groups with each other. For the comparison of physical characteristics and viscoelastic properties between hemophilia severity and the healthy group, one-way ANOVA was used. In all analyses, a *p* value < 0.05 was considered statistically significant.

Small (0–20), medium (20–50), and large (>80) effect sizes (*Cohen*-d) were defined. Effect size can be used to determine the true effectiveness of evaluations and to obtain the power of results.

The power analysis was performed with the use of G*Power software, v. 3.1.9.2, based on the expectation of a large effect size (f = 0.50). The minimum sample size required for comparisons between two groups was calculated as 31. Considering possible data loss, the total number of samples was increased by 20% and calculated as 40, and 20 people were planned to be included in the study for each group (α = 0.05; 1 − β = 0.85).

## 3. Results

A total of 40 individuals, including 20 hemophilia patients with an average age of 11.9 ± 3.95 and 20 healthy children with an average age of 12.6 ± 3.41, were included in the study. Of the 20 hemophilia patients participating in the study, 13 were diagnosed with hemophilia A and 7 with hemophilia B. Twelve patients had moderate-type and eight patients had severe-type hemophilia. Ten patients described a history of a lower-extremity target joint (seven knee joints, three ankle joints) and at least one history of lower-extremity (iliopsoas quadriceps femoris, hamstring, and gastrocinemius) hematoma. Since all children diagnosed during postnatal surgical interventions such as circumcision had severe or moderate hemophilia, they started prophylaxis treatment (Standard Half Life—SHL) at the age of 2, considering the risk of inhibitor development. However, the included patients did not experience acute joint and muscle bleeding in the previous month; patients who received non-prophylaxis concentrated factor therapy were not included in the sample. It was stated that all patients should receive factor treatment early in the day when they wake up in the morning to ensure their compliance with the prophylaxis treatment. Information on the demographic characteristics of individuals is included in Table 1.

When the viscoelastic properties of the dominant-side lower extremity of the individuals participating in the study were compared, it was determined that the vastus lateralis muscle elasticity had a significant difference between the groups (*p* = 0.012) and large effect sizes (d = 0.83), and the results regarding the viscoelastic properties of the other muscles were similar between the two groups (*p* > 0.05). In the hemophilia group, muscle elasticity related to VL on the non-dominant side decreased logarithmically compared to the healthy group (Table 2).

In the evaluation of the viscoelastic properties of the non-dominant-side lower-extremity muscles of the hemophilia group and healthy children, no difference was found between the two groups (*p* > 0.05) (Table 3).

When the viscoelastic properties of patients with moderate and severe hemophilia and healthy children were compared, a difference was found in the elasticity of the dominant-side VL muscle (*p* = 0.032). After an advanced statistical analysis to find the source of the difference in VL muscle elasticity measurements between the groups, it was determined that there was a difference between the patients with severe hemophilia and the healthy group (*p* = 0.041) (Table 4).

## 4. Discussion

This study aimed to evaluate the viscoelastic properties, including tone, stiffness, and elasticity, of the lower-extremity muscles in pediatric hemophilia patients with myotonometric measurements and compare them with their healthy peers. Our study is the first to report the viscoelastic properties of seven different muscles of the lower extremity by bilateral measurements in a pediatric hemophilia group. Forty male individuals between the ages of 6 and 17 were included in the study. Since women are carriers of hemophilia, the healthy group was selected entirely from men in order to eliminate gender differences. The age, height, body weight, and BMI values of the two groups were similar. When the differences between the two groups were examined, it was determined that the elasticity of the vastus lateralis muscle of children with hemophilia was reduced on the dominant side compared to healthy children. When the muscles measured—except the vastus lateralis muscle—were examined in terms of tone, stiffness, and elasticity, the mechanical properties of children with hemophilia and healthy children showed similar results.

In a study conducted by Sakkool et al. on 30 healthy children aged 5–7, the tone, stiffness, and elasticity of the RF, TA, BF and GM muscles were measured. As a result of measurements made on four muscles, it was stated that myotonometric measurements in children showed high reliability. When the measurement results were examined, the viscoelastic values of healthy children were parallel to the healthy group in our study [26]. Havuc et al. measured the tone, stiffness, and elasticity of the GL and GM muscles with MyotonPRO in 40 children with CP between the ages of 2 and 18 and compared them with 20 healthy children. Although the viscoelastic properties of the GL muscle were worse than the healthy group, this did not create a statistical difference between the two groups. In the GM muscle, while elasticity was similar between the two groups, tone and stiffness showed worse results in the CP group. In our study, when we looked at the GM and GL measurement results in the hemophilia group, tone was higher and elasticity was lower, but there were no significant differences between the groups [17]. Hemarthroses occur in the knee in 60% of patients with hemophilia, affecting the biomechanical alignment of the lower extremity [27]. In this context, the tone may be higher than the healthy group, especially in the gastrocnemius muscle group, whose activation is important during standing and walking phases.

According to the studies examining the relationship between BMI and the mechanical properties of muscle, studies conducted with MyotonPRO suggest that increasing BMI changes the mechanical properties of muscle. BMI was similar between the two groups in our study. However, we did not evaluate intramuscular components physiologically. As men have more anthropometric muscle mass and lower fat content, muscle fiber and cross-sectional areas may affect the measurement results [28,29]. For this reason, the fact that both groups were male may not have revealed significant differences in muscle mechanics. It is beneficial to compare the mechanical properties of the muscle by taking anthropometric measurements into consideration in future studies.

Studies with different measurement devices have suggested that BMI has no effect on biomechanical muscle parameters [30,31]. An opposing study linked BMI to differences in upper trapezius muscle stiffness (7.5%) and elasticity (4%) assessed by MyotonPRO. Therefore, using BMI calculated using height and body weight may not be an appropriate method for myotonometric evaluations. A study conducted on sedentary individuals stated that there was an inverse relationship between muscle tone and subcutaneous fat tissue [32]. A thick layer of subcutaneous fat can alter muscle responses, reducing oscillations and frequencies. This may cause a decrease in muscle tone. So, other body composition measures such as subcutaneous fat thickness, lean body mass, and fat mass index could be investigated in future studies to elucidate changes in muscle mechanical properties.

A recent study reported that individuals with plantar fasciitis of the lower extremity had a stiff GM in the gastrocnemius muscle, but no increased stiffness in the GL muscle [33]. Lidström et al. measured the tissue elasticity of the RF muscles of healthy children and children with CP with the Myoton device and compared these values. RF elasticity showed similar results in both groups [34]. In 2022, Nunez et al. examined the viscoelastic properties of the BF muscle in professional football players with and without a history of hamstring injury. Tone, hardness, and elasticity values in injured BF muscles differed compared to BF muscles without any injury history. Based on the study results, acute injuries to the muscle negatively affect the mechanical properties of the muscle [16]. The similarity of the mechanical properties of the BF muscle in both groups in our study can be explained by the fact that our children with hemophilia were selected from patients who did not have a history of acute bleeding in the previous month. Muscle mechanical properties may vary in individuals with a history of acute bleeding. In future studies, examining the mechanical properties after acute muscle bleeding in hemophilia will shed light on the literature.

Gustafson et al. stated that periarticular QF and hamstring muscle stiffness were higher in adult gonarthrosis patients compared to asymptomatic individuals [35]. In diseases such as gonarthrosis, which occur at advanced ages and result in cartilage degeneration due to overuse of the knee, muscle involvement increases with age. In our study, the fact that there was no difference in the healthy group for these muscles, which have an important role in the lower extremity, can be explained by the fact that hemophilia is characterized by hemarthroses at an early age and causes joint problems, but does not directly affect muscle mechanics negatively.

In the study by Ohko et al., stiffness measurements of the RF, VM, and VL muscles of 25 patients diagnosed with knee osteoarthritis and 25 healthy individuals were evaluated with MyotonPRO. Stiffness values of RF and VM muscles showed similar results in both groups, but VL muscle stiffness was found to be higher in individuals with osteoarthritis [36]. Looking at hemophilia patients, the majority of hemarthroses that occur in the lower extremities are seen in the knee. VM and BF measurements in our hemophilia patients, who had a clinical picture similar to osteoarthritis, were found to be similar to the healthy group. However, in our study, while VL muscle stiffness was similar in both groups, decreased elasticity and a high effect level were detected in hemophilia. The frequency of spontaneous bleeding episodes may have resulted in a greater decrease in elasticity in patients with moderate hemophilia compared to the healthy group. As bleeding into the knee joint and surrounding muscles increases with age, the stiffness of the VL muscle may vary. However, the fact that VL may decrease in elasticity due to the deterioration of lower-extremity biomechanics after repeated bleeding into the knee joint is among the strengths of our study. It is important for clinicians to evaluate the elasticity of this muscle in hemophilia patients and include it in treatment programs ensuring knee stabilization.

Our study contributes significantly to the literature as it is the first study to evaluate the viscoelastic properties of lower-extremity muscles in hemophilia patients. However, there are some limitations in our study. If the evaluations of older age groups had been included in the study and a comparison had been made between age-related groups, definitive results could have been presented regarding the variability of early and late muscle mechanics. The lack of an evaluation of an adult hemophilia group in the literature limited our study results. Secondly, if we could objectively evaluate whether the muscles could relax sufficiently in the resting position with devices such as EMG, we could obtain better results, especially in tone variability. Finally, if we had been able to make anthropometric measurements of skin stiffness, skin thickness, and subcutaneous fat tissues, we could have revealed significant differences between the two groups.

## 5. Conclusions

In conclusion, our study is important because it is the first study to provide measurement results of the viscoelastic properties of lower-extremity muscles in patients with hemophilia with MyotonPRO. Decreased elasticity in the VL muscle in children with hemophilia can cause various knee-related problems. Problems in lower-extremity biomechanics, which will result in decreased lateral stabilization of the knee, will trigger recurrent joint bleeding. For this reason, clinicians should include evaluation and treatment options regarding knee stabilization and VL muscle elasticity in rehabilitation processes in order to minimize unavoidable deformities that may occur in older age groups.

## Figures and Tables

**Table 1:** Descriptive characteristics of the participants according to the groups.

Variables	Hemophilia Group (*n* = 20)		Healthy Group (*n* = 20)			
	X ± SD	(Min–Max)	X ± SD	(Min–Max)	t	*p*
Age (y)	11.9 ± 3.95	6–17	12.6 ± 3.41	6–17	−0.60	0.552
Height (cm)	1.46 ± 2.64	1−1.8	1.53 ± 1.73	1.12–1.8	−0.55	0.584
Body mass (kg)	40.50 ± 17.09	14–72	44.40 ± 17.17	18–79	−0.72	0.476
BMI (kg/m^2^)	17.89 ± 2.79	13.20–23.80	18.88 ± 3.76	14.30–28.70	−0.95	0.351

**Table 2:** Comparison of viscoelastic properties of dominant-side lower-extremity muscles between groups.

Parameter	Hemophilia Group (*n* = 20) (X ± SD)	Healthy Group (*n* = 20) (X ± SD)	t	*p*	*Cohen* d
VMO tone (Hz)	13.25 ± 0.68	13.04 ± 1.18	0.67	0.506	0.21
VMO stiffness (N/m)	210.45 ± 28.27	200.99 ± 40.13	0.86	0.394	0.27
VMO elasticity (log)	1.01 ± 0.15	0.96 ± 0.23	0.85	0.402	0.27
RF tone (Hz)	13.90 ± 0.96	13.84 ± 1.39	0.17	0.864	0.05
RF stiffness (N/m)	223.70 ± 33.94	230.87 ± 42.55	−0.59	0.560	0.19
RF elasticity (log)	1.08 ± 0.22	1.08 ± 0.21	−0.01	0.994	0.00
VL tone (Hz)	14.13 ± 1	14.78 ± 1.48	−1.63	0.112	0.51
VL stiffness (N/m)	246.77 ± 32.45	260.30 ± 41.90	−1.14	0.261	0.36
VL elasticity (log)	1.08 ± 0.19	1.24 ± 0.19	2.63	**0.012**	**0.83**
BF tone (Hz)	14.34 ± 0.98	14.25 ± 3.34	0.12	0.909	0.04
BF stiffness (N/m)	229.54 ± 37.35	245.95 ± 32.65	−1.48	0.147	0.47
BF elasticity (log)	1.09 ± 0.20	1.08 ± 0.18	0.20	0.842	0.06
TA tone (Hz)	17.49 ± 1.81	17.72 ± 1.79	−0.41	0.682	0.13
TA stiffness (N/m)	351.70 ± 52.72	343.06 ± 52.14	0.52	0.605	0.16
TA elasticity (log)	0.80 ± 0.10	0.77 ± 0.12	0.76	0.455	0.24
GL tone (Hz)	15.06 ± 1.16	14.90 ± 1.69	0.36	0.721	0.11
GL stiffness (N/m)	259.04 ± 29.88	253.28 ± 44.06	0.48	0.632	0.15
GL elasticity (log)	1.11 ± 0.16	1.03 ± 0.17	1.58	0.122	0.50
GM tone (Hz)	14.30 ± 1.33	14.20 ± 1.35	0.25	0.806	0.08
GM stiffness (N/m)	234.40 ± 28.46	237.17 ± 32.95	−0.29	0.777	0.09
GM elasticity (log)	1.14 ± 0.17	1.12 ± 0.20	0.41	0.686	0.13

VMO—vastus medialis obliquus, RF—rectus Femoris, VL—vastus lateralis, BF—biceps femoris, TA—tibialis anterior, GL—gastrocinemius lateralis, GM—gastrocinemius medialis. Bold denotes statistically significant values.

**Table 3:** Comparison of viscoelastic properties of non-dominant-side lower-extremity muscles between groups.

Parameter	Hemophilia Group (*n* = 20) (X ± SD)	Healthy Group (*n* = 20) (X ± SD)	t	*p*	*Cohen* d
VMO tone (Hz)	13.11 ± 0.91	12.91 ± 0.91	0.70	0.489	0.22
VMO stiffness (N/m)	206.31 ± 35.93	199.83 ± 36.34	0.57	0.574	0.18
VMO elasticity (log)	1.03 ± 0.15	0.98 ± 0.26	0.65	0.519	0.21
RF tone (Hz)	13.60 ± 0.79	13.74 ± 1.23	−0.41	0.682	0.13
RF stiffness (N/m)	218.90 ± 27.68	226.64 ± 34.05	−0.79	0.435	0.25
RF elasticity (log)	0.99 ± 0.14	1.08 ± 0.18	−1.61	0.115	0.51
VL tone (Hz)	14.19 ± 1.41	14.38 ± 1.42	−0.44	0.665	0.14
VL stiffness (N/m)	243.42 ± 38.50	247.87 ± 31.95	−0.40	0.693	0.13
VL elasticity (log)	1.22 ± 0.16	1.15 ± 0.33	0.87	0.388	0.28
BF tone (Hz)	14.32 ± 1.10	14.58 ± 1.27	−0.70	0.486	0.22
BF stiffness (N/m)	232.35 ± 41.67	239.05 ± 31.92	−0.57	0.571	0.18
BF elasticity (log)	1.09 ± 0.20	1.07 ± 0.19	0.34	0.735	0.11
TA tone (Hz)	18.03 ± 1.59	17.78 ± 1.99	0.43	0.669	0.14
TA stiffness (N/m)	366.16 ± 63.24	349.26 ± 60.82	0.86	0.395	0.27
TA elasticity (log)	0.96 ± 0.67	0.78 ± 0.09	1.16	0.254	0.37
GL tone (Hz)	15.09 ± 1.37	15.04 ± 1.92	0.09	0.932	0.03
GL stiffness (N/m)	259.10 ± 34.83	259.52 ± 44.25	−0.03	0.974	0.01
GL elasticity (log)	1.07 ± 0.15	1.03 ± 0.24	0.71	0.483	0.22
GM tone (Hz)	14.52 ± 1.10	14.21 ± 1.26	0.83	0.412	0.26
GM stiffness (N/m)	235.23 ± 23.73	232.96 ± 27.33	0.28	0.780	0.09
GM elasticity (log)	1.12 ± 0.19	1.12 ± 0.16	0.07	0.944	0.02

VMO—vastus medialis obliquus, RF—rectus Femoris, VL—vastus lateralis, BF—biceps femoris, TA—tibialis anterior, GL—gastrocinemius lateralis, GM—gastrocinemius medialis.

**Table 4:** Comparison of physical characteristics and viscoelastic properties between hemophilia severity and healthy group.

Variables	Moderate-Severity Group X ± SD	Severe-Severity Group X ± SD	Healthy Group X ± SD	f	*p*
Age (y)	11.92 ± 4.32	11.88 ± 3.60	12.6 ± 3.40	0.176	0.840
Height (cm)	145.25 ± 30.15	148.25 ± 21.52	150.35 ± 17.34	0.191	0.827
Body mass (kg)	40.33 ± 18.58	40.75 ± 15.0	44.4 ± 17.16	0.254	0.777
BMI (kg/m^2^)	17.87 ± 2.56	17.9 ± 3.29	18.87 ± 3.76	0.435	0.651
RVMO tone (Hz)	13.42 ± 0.50	12.97 ± 0.85	13.04 ± 1.18	0.749	0.480
RVMO stiffness (N/m)	217.44 ± 23.70	199.96 ± 32.2	200.99 ± 40.13	0.986	0.383
RVMO elasticity (log)	1.04 ± 0.16	0.96 ± 0.12	0.96 ± 0.22	0.767	0.472
LVMO tone (Hz)	13.34 ± 0.98	12.76 ± 0.68	12.91 ± 0.90	1.257	0.296
LVMO stiffness (N/m)	215.25 ± 38.16	192.87 ± 29.52	199.83 ± 36.33	1.107	0.341
LVMO elasticity (log)	1.05 ± 0.17	0.97 ± 0.12	0.98 ± 0.25	0.6	0.554
RRF tone (Hz)	13.88 ± 0.84	13.92 ± 1.18	13.83 ± 1.38	0.017	0.983
RRF stiffness (N/m)	227.69 ± 24.18	217.71 ± 46.25	230.86 ± 42.55	0.329	0.722
RRF elasticity (log)	1.04 ± 0.16	1.13 ± 0.29	1.07 ± 0.21	0.424	0.658
LRF tone (Hz)	13.72 ± 0.77	13.41 ± 0.84	13.73 ± 1.23	0.299	0.743
LRF stiffness (N/m)	224.16 ± 25.95	210.98 ± 30.03	226.63 ± 34.04	0.742	0.483
LRF elasticity (log)	1.00 ± 0.16	0.97 ± 0.11	1.07 ± 0.18	1.337	0.275
RVL tone (Hz)	14.16 ± 1.06	14.07 ± 0.97	14.78 ± 1.47	1.301	0.284
RVL stiffness (N/m)	251.08 ± 30.08	240.28 ± 36.82	260.3 ± 41.90	0.838	0.441
RVL elasticity (log)	1.21 ± 0.22	1.28 ± 0.15	1.08 ± 0.19	3.793	*0.032
LVL tone (Hz)	14.72 ± 1.37	13.37 ± 1.09	14.38 ± 1.41	2.51	0.095
LVL stiffness (N/m)	252.21 ± 37.45	230.21 ± 38.57	247.86 ± 31.94	1.031	0.367
LVL elasticity (log)	1.23 ± 0.15	1.19 ± 0.17	1.14 ± 0.32	0.449	0.641
RBF tone (Hz)	14.42 ± 0.80	14.21 ± 1.26	14.25 ± 3.33	0.024	0.976
RBF stiffness (N/m)	234.03 ± 32.79	222.8 ± 44.84	245.95 ± 32.65	1.322	0.279
RBF elasticity (log)	1.10 ± 0.21	1.08 ± 0.18	1.08 ± 0.18	0.048	0.953
LBF tone (Hz)	14.40 ± 1.10	14.17 ± 1.16	14.58 ± 1.27	0.332	0.719
LBF stiffness (N/m)	239.52 ± 36.58	221.58 ± 48.87	239.05 ± 31.91	0.726	0.491
LBF elasticity (log)	1.12 ± 0.17	1.05 ± 0.24	1.07 ± 0.18	0.363	0.698
RTA tone (Hz)	17.29 ± 2.21	17.77 ± 1.03	17.72 ± 1.78	0.254	0.777
RTA stiffness (N/m)	341.52 ± 45.96	366.95 ± 61.49	343.05 ± 52.14	0.703	0.502
RTA elasticity (log)	0.79 ± 0.12	0.80 ± 0.07	0.77 ± 0.11	0.328	0.722
LTA tone (Hz)	17.9 ± 1.27	18.21 ± 2.05	17.78 ± 1.99	0.161	0.852
LTA stiffness (N/m)	357.31 ± 45.88	379.41 ± 84.92	349.26 ± 60.81	0.668	0.519
LTA elasticity (log)	0.84 ± 0.10	1.12 ± 1.07	0.78 ± 0.08	1.562	0.223
RGL tone (Hz)	15.53 ± 1.14	14.35 ± 0.81	14.89 ± 1.69	1.766	0.185
RGL stiffness (N/m)	260.97 ± 31.69	256.12 ± 28.80	253.28 ± 44.06	0.153	0.859
RGL elasticity (log)	1.12 ± 0.14	1.08 ± 0.19	1.02 ± 0.17	1.421	0.254
LGL tone (Hz)	15.37 ± 1.40	14.65 ± 1.28	15.04 ± 1.91	0.456	0.637
LGL stiffness (N/m)	263.85 ± 37.29	251.96 ± 31.80	259.51 ± 44.24	0.211	0.810
LGL elasticity (log)	1.07 ± 0.17	1.05 ± 0.13	1.02 ± 0.24	0.272	0.763
RGM tone (Hz)	14.67 ± 1.39	13.73 ± 1.08	14.19 ± 1.35	1.246	0.299
RGM stiffness (N/m)	240.32 ± 30.68	225.5 ± 23.86	237.17 ± 32.95	0.599	0.555
RGM elasticity (log)	1.16 ± 0.20	1.09 ± 0.07	1.11 ± 0.19	0.433	0.652
LGM tone (Hz)	14.62 ± 1.33	14.35 ± 0.66	14.20 ± 1.26	0.465	0.632
LGM stiffness (N/m)	237.85 ± 25.56	231.28 ± 21.74	232.95 ± 27.33	0.194	0.824
LGM elasticity (log)	1.13 ± 0.20	1.11 ± 0.20	1.12 ± 0.16	0.016	0.984

* *p* < 0.05, one-way ANOVA test results, VMO—vastus medialis obliquus, RF—rectus femoris, VL—vastus lateralis BF—biceps femoris, TA—tibialis anterior, GL—gastrocinemius lateralis, GM—gastrocinemius medialis. R-right, L-left. Bold denotes statistically significant values.

## Data Availability

For the complete database, please contact us by email: serkan.usgu@hku.edu.tr. Data are not publicly available due to privacy and ethical restrictions.
